# Development and validation of prognostic model for predicting mortality of COVID-19 patients in Wuhan, China

**DOI:** 10.1038/s41598-020-78870-6

**Published:** 2020-12-31

**Authors:** Qi Mei, Amanda Y. Wang, Amy Bryant, Yang Yang, Ming Li, Fei Wang, Jia Wei Zhao, Ke Ma, Liang Wu, Huawen Chen, Jinlong Luo, Shangming Du, Kathrin Halfter, Yong Li, Christian Kurts, Guangyuan Hu, Xianglin Yuan, Jian Li

**Affiliations:** 1 Tongji Hospital, Tongji Medical College, Huazhong University of Science and Technology, Wuhan, Hubei People’s Republic of China; 2 The Renal and Metabolic Division, The George Institute for Global Health, University of New South Wales, Sydney, Australia; 3 Concord Clinical School, The University of Sydney, Sydney, Australia; 4 Department of Renal Medicine, Concord Repatriation General Hospital, Concord, Australia; 5 Department of Biomedical and Pharmaceutical Sciences, College of Pharmacy, Idaho State University, Meridian, ID USA; 6 Renmin Hospital of Wuhan University, Wuhan, Hubei People’s Republic of China; 7 Department of Respiratory and Critical Care Medicine, Wuhan Pulmonary Hospital, Wuhan, Hubei People’s Republic of China; 8 Department of Chinese Medicine, Wuhan No.1 Hospital, Tongji Medical College, Huazhong University of Science and Technology, Wuhan, Hubei People’s Republic of China; 9 The Faculty of Pharmacy, The University of Sydney, Sydney, Australia; 10 Department and Institute of Infectious Disease, Tongji Hospital, Tongji Medical College, Huazhong University of Science and Technology, Wuhan, Hubei People’s Republic of China; 11 Department of Emergency Medicine, Tongji Hospital, Tongji Medical College, Huazhong University of Science and Technology, Wuhan, Hubei People’s Republic of China; 12 Institute for Medical Informatics, Biometry and Epidemiology, Ludwig-Maximilians-University Munich, Munich, Germany; 13 Department of Respiratory and Critical Care Medicine, National Clinical Research Center of Respiratory Disease, Tongji Hospital, Tongji Medical College, Huazhong University of Science and Technology, Wuhan, Hubei People’s Republic of China; 14 Institute of Experimental Immunology, University Clinic of Rheinische Friedrich-Wilhelms-University, Bonn, Germany

**Keywords:** Infectious diseases, Respiratory tract diseases, Biomarkers, Health care, Risk factors

## Abstract

Novel coronavirus 2019 (COVID-19) infection is a global public health issue, that has now affected more than 200 countries worldwide and caused a second wave of pandemic. Severe adult respiratory syndrome-CoV-2 (SARS-CoV-2) pneumonia is associated with a high risk of mortality. However, prognostic factors predicting poor clinical outcomes of individual patients with SARS-CoV-2 pneumonia remain under intensive investigation. We conducted a retrospective, multicenter study of patients with SARS-CoV-2 who were admitted to four hospitals in Wuhan, China from December 2019 to February 2020. Mortality at the end of the follow up period was the primary outcome. Factors predicting mortality were also assessed and a prognostic model was developed, calibrated and validated. The study included 492 patients with SARS-CoV-2 who were divided into three cohorts: the training cohort (n = 237), the validation cohort 1 (n = 120), and the validation cohort 2 (n = 135). Multivariate analysis showed that five clinical parameters were predictive of mortality at the end of follow up period, including advanced age [odds ratio (OR), 1.1/years increase (*p* < 0.001)], increased neutrophil-to-lymphocyte ratio [(NLR) OR, 1.14/increase (*p* < 0.001)], elevated body temperature on admission [OR, 1.53/°C increase (*p* = 0.005)], increased aspartate transaminase [OR, 2.47 (*p* = 0.019)], and decreased total protein [OR, 1.69 (*p* = 0.018)]. Furthermore, the prognostic model drawn from the training cohort was validated with validation cohorts 1 and 2 with comparable area under curves (AUC) at 0.912, 0.928, and 0.883, respectively. While individual survival probabilities were assessed, the model yielded a Harrell’s C index of 0.758 for the training cohort, 0.762 for the validation cohort 1, and 0.711 for the validation cohort 2, which were comparable among each other. A validated prognostic model was developed to assist in determining the clinical prognosis for SARS-CoV-2 pneumonia. Using this established model, individual patients categorized in the high risk group were associated with an increased risk of mortality, whereas patients predicted to be in the low risk group had a higher probability of survival.

## Introduction

Novel coronavirus (COVID-19) infection is a global public health issue that has now affected more than 200 countries worldwide and caused second wave of pandemic^1^. Severe adult respiratory syndrome-CoV-2 (SARS-CoV-2) pneumonia is associated with a high risk of mortality. However, factors that predict poor clinical outcomes of individual patients with SARS-CoV-2 pneumonia remains under intensive investigation.

Current studies have showed that patients with SARS-CoV-2 pneumonia exhibit a wide range of symptoms such as fever, cough, myalgia, fatigue, or others^2–5^. Many patients experience a mild disease course, although approximately 15–25% develop more severe disease. Progression may result in acute respiratory distress syndrome (ARDS), multiple organ failure, and death^6^. Therefore, it is of ultimate importance to identify the high-risk group of patients in order to implement prompt medical intervention to improve clinical outcomes.

The aim of this study was to establish and validate a prognostic model for increased risk of mortality and survival time among individual patients with SARS-CoV-2 pneumonia. Our validated model stratifies patients into those with high versus low risk of death before life-threatening complications develop. This knowledge could be used to inform and justify critical patient management decisions and promote optimal use of often limited medical resources during the COVID-19 pandemic.

## Methods

### Study design and participants

This retrospective, multi-center cohort study involved adults patients who were diagnosed with COVID-19 pneumonia in four major government designated hospitals in Wuhan: Tongji Hospital of Tongji Medical College of Huazhong University of Science and Technology (TJH), Renmin Hospital of Wuhan University (RHWU), Wuhan Pulmonary Hospital (WPH), and Wuhan No.1 Hospital of Tongji Medical College of Huazhong University of Science and Technology (WNH) (Supplementary Fig. 1). Patients were followed until the 18th September, 2020. Patients were divided into three cohorts: the training cohort (TC) was used for establishment of a prognostic model, and 2 validation cohorts (VC1 and VC2) were used for external validation and assessment of robustness of the models. The TC included data collected from TJH between Jan 21th and Feb 16th, 2020. The VC1 consisted of patients from RHW and WNH admitted between Jan 23rd and Feb 16th, 2020. The VC2 included patients from WPH admitted between Jan 10th and Feb 27th, 2020. The primary outcome was mortality at the end of the study period. To be included for study, participants had to meet following diagnostic criteria for COVID-19 pneumonia: (1) confirmed diagnosis of SARS-CoV-2 pneumonia using RT-PCR on nasopharyngeal/oropharyngeal swab samples , (2) Computerised tomography (CT) evidence of viral pneumonia, defined as COVID-19. In addition, patient clinical outcome data had to be available. Exclusion criteria included: (1) death occurred within 24 h after hospital admission and for which related health records were unavailable, (2) no data on clinical outcomes were available, (3) suspected cases lacked a positive result for F137nCoV test, and (4) patients refused to participate in this study.

Following informed consent, the following data were collected on admission:age, sex, symptoms from onset to hospital admission (fever, cough, dyspnea, myalgia, rhinorrhea, arthralgia, chest pain, headache, and vomiting), comobidities (cardiovascular disease, chronic pulmonary disease, cerebrovascular disease and chronic neurological disorders, diabetes, malignancy, and smoking), vital signs (heart rate, respiratory rate, and blood pressure), laboratory values on admission (serum hemoglobin concentration, lymphocyte counts, platelet counts, diverse protein markers), treatment regime used for COVID-19 pneumonia (antiviral agents, antibacterial agents, corticosteroids, and interferon therapy), dates of symptom onset, admission, virus testing, CT-scan, as well as changes in patient condition and living status. All methods were carried out in accordance with relevant guidelines and regulation in Declaration of Helsinki.

### Treatment protocol and criteria for discharged from hospitals for SARS-CoV-2 pneumonia

The treatment strategy for patients with COVID-19 pneumonia was based on the guidelines of World Health Organization (WHO)^7^, and included symptoms relief, treatment of underlying diseases, prevention of superimposed bacterial infections, active prevention of complications such as sepsis and ARDS and timely support of vital organ function. Oxygen supplementation was provided for patients with reduced O2 saturations and was administered via high flow oxygen via nasal prong (< 300 mmHg), non-invasive and invasive mechanical ventilation (< 200 and < 150 mmHg, respectively), or extracorporeal membrane oxygenation (ECMO) if required.

The discharge criteria for patients with SARS-CoV-2 pneumonia included one of the following: (1) haemodynamically stable and afebrile for > 3 days, (2) radiological evidence of significant resolution of pneumonia on CT-scan, (3) two sequential negative results for the F137nCoV test with at least 1 day interval, and (4) no concurrent acute medical issues requiring transfer to another medical facility.

### Statistical considerations

Survival time was calculated from the date of hospital admission until death due to SARS-CoV-2 pneumonia or until the date of the last follow-up. Death due to SARS-CoV-2 pneumonia was considered as an event. Continuous variables were reported as means with standard deviations (SD) for normally distributed variables and as medians and interquartile ranges (IQR) for non-normally distributed variables. Categorical variables were reported as proportion.

According to the transparent reporting of a Multivariable Prediction Model for Individual Prognosis or Diagnosis guidelines^8^, we developed a model using the training and validation cohorts. During the model development, all patients’ demographic characteristics, clinical information, vital signs and laboratory values were analyzed for a possible association between the life status (deceased versus living) and survival time using the least absolute shrinkage and selection operator (LASSO) for multivariable selection^9^. An iterative process combining forward and backward selection was applied to remove non-significant covariates. During each step of the iteration, the Akaike information criterion (AIC) was used to evaluate model fit^10^. The final model was then established with a minimum value of AIC. The AUC value was used to evaluate the accuracy of the prediction for the vital status. Model calibration was performed to ensure the robustness. The Cox proportional hazards regression analysis was used to evaluate the assessment of the prognostic model for individual survival times. Proportional hazards assumption for the Cox proportional hazards regression model was assessed by using the Schoenfeld residuals test.

In order to validate the prognostic model, two independent validation cohorts (VC1, VC2) with the same discrimination method and survival function were used. The 95% confidence intervals (CIs) were estimated via 5000 bootstraps replicates. All statistical analyses were performed using R software version 3.6. A *p* < 0.05 was considered as statistically significant.

### Role of funding

The funders were not involved in any activities of this study, aside from providing financing.

### Ethics, consent and permission

This study was approved by the institutional ethics board of Tongji Hospital of Tongji Medical College, Huazhong University of Science and Technology (No. IRBID:TJ-C 20,200,107). All participants agreed to take part in this study.

### Consent to publish

Informed consents were obtained from participants for the purpose of publication.

## Results

### Demographic and clinical features in training and validation cohorts

Overall 492 patients were recruited in this study. The demographic characteristics and clinical features of patients from the TC (n = 237; TJH), VC1 (n = 120; RHWU + WNH), and VC2 (n = 135; WPH) cohorts are listed in the Table 1. The mortality rates in the three cohorts were 44.3% (TC), 25.8% (VC1) and 33.3% (VC2), respectively. A total of 105 events occurred in the TC, events in VC1 and VC2 were 31 and 45, respectively. The median survival times were comparable among these three cohorts (15, 17 and 14 days for TC, VC1, and VC2, respectively). TC patients had a median age of 62 (IQR 50–70) and were older than those in VC1 (median age 46, IQR 37–66), but similar to those in VC2 (median age 63, IQR 52–70.5). There was no significant difference in sex distribution among the three cohorts. Most of patients were non-smokers (92% [TC], 97.5% [VC1], and 83% [VC2]). The number of patients with associated comorbidities varied between three cohorts (52.7% [TC] vs. 37.5% [VC1] vs. 73.3% [VC2]; Table 1). The number of severe cases requiring intensive care unit (ICU) admission also varied among the three cohorts (8.9% [TC], 1.7% [VC1], and 20% [VC2]). Lymphopenia occurred in the majority of patients (69.2% [TC], 60.8% [VC1], and 75.6% [VC2], Table 1). Leucocytosis was observed 24.5% in the TC, 21.7% in the VC1, and 20.7% in theVC2. Neutrophilia was observed in 34.6% in the TC, 30.8% in the VC1, and 31.1% in the VC2.

**Table 1:** Clinical characteristics, treatments and laboratory findings of the Training (TJH), Validation 1 (RHWU + WNH), and Validation 2 (WPH) cohorts.

	Overall	Training cohort (TJH)	Validation 1 (RHWU + WNH)	Validation 2 (WPH)
**Characteristics**				
Number of patients	492	237	120	135
Median age, years	61.0 (45.0–70.0)	62 (50.0–70.0)	46.0 (37.0–66.0)	63.0 (52.0–70.5)
Survival outcome	…	…	…	…
Cured	311 (63.2)	132 (55.7)	89 (74.2)	90 (66.7)
Deceased	181 (36.8)	105 (44.3)	31 (25.8)	45 (33.3)
Sex	…	…	…	…
Male	221 (44.9)	100 (42.2)	52 (43.3)	69 (51.1)
Female	271 (55.1)	137 (57.8)	68 (56.7)	66 (48.9)
ICU care	…	…	…	…
Yes	50 (10.2)	21 (8.9)	2 (1.7)	27 (20.0)
No	442 (89.8)	216 (91.1)	118 (98.3)	108 (80.0)
Smoking	…	…	…	…
Yes	45 (9.1)	19 (8.0)	3 (2.5)	23 (17.0)
No	447 (90.9)	218 (92.0)	117 (97.5)	112 (83.0)
Comorbidities	269 (54.7)	125 (52.7)	45 (37.5)	99 (73.3)
Hypertension	161 (32.7)	83 (35.1)	27 (22.5)	51 (37.8)
Diabetics	74 (15.0)	38 (16.0)	11 (9.2)	25 (18.5)
CVDs	42 (8.5)	25 (10.5)	5 (4.2)	12 (8.9)
Carcinoma	18 (3.7)	7 (3.0)	3 (2.5)	8 (6.3)
Initial common symptom	…	…	…	…
Fever	329 (66.9)	174 (73.4)	73 (60.8)	82 (60.7)
Cough	270 (54.9)	136 (57.4)	62 (51.7)	71 (53.3)
Myalgia or fatigue	185 (37.6)	93 (39.2)	35 (29.2)	57 (42.2)
Dyspneu	137 (28.3)	76 (32.1)	26 (21.7)	37 (27.4)
Admission body temperature, °C	36.0 (36.5–37.5)	36.9 (36.5–37.8)	36.8 (36.5–37.4)	36.6 (36.4–36.9)
Symptom onset to admission, days	9.0 (6.0–12.0)	10 (7.0–14.0)	7.0 (4.0–10.0)	10.0 (7.0–13.0)
Hospitalization, days	15.0 (9.8–23.0)	15 (7.0–24.0)	17.0 (11.8–25.0)	14.0 (10.0–19.0)
Systolic pressure, mm Hg	126.0 (116.0–140.0)	130 (119.0–143.0)	124.5 (117.0–136.0)	125.0 (113.0–135.0)
Respiratory rate, breaths per min	20.0 (20.0–24.0)	20 (20.0–24.0)	20.0 (19.8–22.0)	21.0 (20.0–25.0)
Pulse rate, beats per min	89.0 (80.0–101.0)	89 (78.0–103.0)	90.0 (78.8–100.0)	87.0 (80.0–98.0)
**Treatments**				
Therapy	…	…	…	…
Antibiotics	432 (87.8)	205 (86.5)	111 (92.5)	116 (85.9)
Antiviral treatment	481 (97.8)	235 (99.6)	115 (95.8)	131 (97.0)
Corticosteroids	338 (68.7)	150 (63.3)	82 (68.3)	106 (78.5)
Interferon treatment	238 (48.4)	89 (37.6)	27 (22.5)	122 (90.4)
Immunoglobin	179 (36.4)	78 (32.9)	27 (22.5)	74 (54.8)
Oxygen therapy	376 (76.4)	200 (84.4)	90 (75.0)	86 (63.7)
Nasal catheter inhalation	390 (79.3)	204 (86.1)	92 (76.7)	94 (69.6)
NIMV	141 (28.7)	95 (40.1)	28 (23.3)	18 (13.3)
IMV	58 (11.8)	27 (11.4)	9 (7.5)	22 (16.3)
ECMO	10 (2.0)	1 (0.4)	1 (0.8)	8 (5.9)
**Laboratory findings**				
WBC count, × 10^9^/L	…	…	…	…
Decrease	69 (14.0)	28 (11.8)	20 (16.7)	21 (15.6)
Normal range	311 (63.2)	151 (63.7)	71 (59.2)	86 (63.7)
Increase	112 (22.8)	58 (24.5)	26 (21.7)	28 (20.7)
Neutrophil count, × 10^9^/L	…	…	…	…
Decrease	51 (10.4)	16 (6.8)	23 (19.2)	12 (8.9)
Normal range	280 (56.9)	139 (58.6)	60 (50.0)	81 (60.0)
Increase	161 (32.7)	82 (34.6)	37 (30.8)	42 (31.1)
Lymphocyte count, × 10^9^/L	…	…	…	…
Decrease	339 (68.9)	164 (69.2)	73 (60.8)	102 (75.6)
Normal range	153 (31.1)	73 (30.8)	47 (39.2)	33 (24.4)
PLT count, × 10^9^/L	…	…	…	…
Decrease	80 (19.0)	47 (19.8)	13 (10.8)	20 (14.8)
Normal range	320 (76.0)	178 (75.1)	31 (25.8)	111 (82.2)
Increase	21 (5.0)	12 (5.1)	5 (4.2)	4 (3.0)
APTT, s	…	…	…	…
Decrease	58 (11.8)	13 (5.5)	34 (28.3)	11 (8.1)
Normal range	345 (70.1)	161 (67.9)	77 (64.2)	107 (79.3)
Increase	89 (18.1)	63 (26.6)	9 (7.5)	17 (12.6)
PT, s	…	…	…	…
Normal range	360 (151.3)	150 (168.5)	109 (403.7)	101 (82.8)
Increase	132 (55.5)	87 (97.8)	11 (40.7)	34 (27.9)
D-D dimer, μg/mL FEU	…	…	…	…
Normal range	206 (86.6)	63 (70.8)	72 (266.7)	71 (58.2)
Increase	286 (120.2)	174 (195.5)	48 (177.8)	64 (52.5)
ALT, U/L	…	…	…	…
Normal range	365 (74.2)	173 (73.0)	95 (79.2)	97 (71.9)
Increase	127 (25.8)	64 (27.0)	25 (20.8)	38 (28.1)
AST, U/L	…	…	…	…
Normal range	311 (63.2)	140 (59.1)	87 (72.5)	84 (62.2)
Increase	181 (36.8)	97 (40.9)	33 (27.5)	51 (37.8)
LDH, U/L	…	…	…	…
Normal range	128 (26.0)	47 (19.8)	57 (47.5)	24 (17.8)
Increase	364 (74.0)	190 (80.2)	63 (52.5)	111 (82.2)
ALP, U/L	…	…	…	…
Decrease	27/421 (6.4)	5 (2.1)	2/49 (4.1)	20 (14.8)
Normal range	351/421 (83.4)	208 (87.8)	39/49 (79.6)	104 (77.0)
Increase	43/421 (10.2)	24 (10.1)	8/49 (16.3)	11 (8.1)
γ-GT, U/L	…	…	…	…
Decrease	2/421 (0.5)	1 (0.4)	0	1 (0.7)
Normal range	324/421 (77.0)	182 (76.8)	39/49 (79.6)	103 (76.3)
Increase	95/421 (22.6)	54 (22.8)	10/49 (20.4)	31 (23.0)
Urea, mmol/L	…	…	…	…
Decrease	33/421 (7.8)	20 (8.4)	3/49 (6.1)	10 (7.4)
Normal range	294/421 (69.8)	166 (70.0)	23/49 (46.9)	105 (77.8)
Increase	94/421 (22.3)	51 (21.5)	23/49 (46.9)	20 (14.8)
Albumin, g/L	…	…	…	…
Decrease	229 (46.5)	139 (58.6)	38 (31.7)	52 (38.5)
Normal range	263 (53.5)	98 (41.4)	82 (68.3)	83 (61.5)
Total cholesterol, mmol/L	…	…	…	…
Normal range	247/266 (92.9)	210/222 (94.6)	6/7 (85.7)	31/37 (83.8)
Increase	19/266 (7.1)	12/222 (5.4)	1/7 (14.3)	6/37 (16.2)
Total bilirubin, μmol/L	…	…	…	…
Normal range	454 (92.3)	219 (92.4)	114 (95.0)	121 (89.6)
Increase	38 (7.7)	18 (7.6)	6 (5.0)	14 (10.4)
hs-CRP, mg/L	…	…	…	…
Normal range	114 (23.2)	54 (22.8)	43 (35.8)	17 (12.6)
Increase	378 (76.8)	183 (77.2)	77 (64.2)	118 (87.4)

Data are median (IQR), n (%), or n/N (%), where N is the total number of patients with available data. ICU = Intensive care unit; (N)IMV = (Non-) Invasive mechanical ventilation; ECMO = Extracorporeal membrane oxygenation; WBC = White blood cell; PLT = Blood platelet; APTT = Activated partial thromboplastin time; PT = Prothrombin time; ALT = Alanine aminotransferase; AST = Aspartate aminotransferase; LDH = Lactate dehydrogenase; ALP = Alkaline phosphatase; γ-GT = gamma-Glutamyl transpeptidase; hs-CRP = high-sensitivity C-reactive protein.

The median time from symptom onset to hospital admission varied between cohorts (TC, median 10.0 days, IQR 7–14 days; VC1, median 7.0 days, IQR 4–10 days; VC2, median 10.0 days, IQR 7–13 days) with fever being the most common symptom on admission. The duration of hospitalization and treatment for TC, VC1, and VC2 were 15.0 days (IQR 7–24 days), 17.0 days (IQR 11.8–25.0 days), and 14.0 days (IQR 10.0–19.0 days), respectively. The majority of patients in TC, VC1, and VC2 were treated with antibiotics (86.5%, 92.5, and 85.9%, respectively) and antivirals (lopinavir/ritonavir; 99.6%, 95.8% and 97.0%, respectively).

### Potential risk factors associated with vital status for COVID-19

Univariate analysis revealed that advanced age, increased body temperature on admission, and the presence of underlying diseases were associated with a higher mortality rate in patients with COVID-19 infection (Table 2). Tachypnoea and hypertension, as well as treatment with antibiotics, corticosteroids or intravenous immunoglobulin were also associated with increased mortality (Table 2). Several laboratory parameters including serum bilirubin, D-dimer, potassium level, prothrombin time (s), lactate dehydrogenase, aspartate transaminase (AST), and urea were also found to be associated with increased risk of death. In addition, patients with lymphopenia, leukocytosis, or neutrophilia also had an increased risk of death (Table 2). Of note, the deceased lymphocyte count, and increased neutrophile count, as well as the increased NLR were also significant risk factors for mortality.

**Table 2:** Risk factors associated with mortality of COVID-19.

Univariate analysis	AOR (95%CI)	Wald’s *p* value
**Demographic and clinical characteristics**		
Median age, years*	1.10 (1.07–1.14)	< .001
Comorbidity: 1 *vs.* 0	2.94 (1.68–5.14)	< .001
No. of comorbidities*	1.72 (1.31–2.26)	< .001
Hypertension: 1 *vs.* 0	2.87 (1.62–5.10)	< .001
Diabetics: 1 *vs.* 0	2.23 (1.04–4.79)	0.039
Admission body temperature, °C*	1.32 (0.99–1.77)	0.030
Systolic pressure, mmHg*	1.03 (1.01–1.04)	< .001
Respiratory rate, breaths per min*	1.17 (1.10–1.25)	< .001
Pulse rate, beats per min*	1.04 (1.02–1.06)	< .001
**Treatments**		
Antibiotics: 1 *vs.* 0	7.80 (2.28–26.65)	0.001
Corticosteroids ≥ 60 mg/day: 1 *vs.* 0	7.45 (3.63–15.31)	< .001
Interferon treatment: 1 *vs.* 0	0.40 (0.22–0.71)	0.002
Immunoglobulin: 1 *vs.* 0	2.82 (1.56–5.10)	< .001
Nasal catheter inhalation: 1 *vs.* 0	3.97 (1.72–9.17)	0.001
Noninvasive mechanical ventilation (NIMV): 1 *vs.*0	370.36 (105.14–1304.61)	< .001
**Immune components**		
White blood cell (WBC) count, × 10^9^/L*	1.38 (1.24–1.53)	< .001
Neutrophil count, × 10^9^/L*	1.45 (1.29–1.63)	< .001
Lymphocyte count, × 10^9^/L *	0.07 (0.03–0.16)	< .001
Neutrophil ratio*	1.39 (1.25–1.46)	< .001
Lymphocyte ratio*	0.18 (0.06–0.25)	< .001
Neutrophil/lymphocyte ratio*	1.19 (1.13–1.26)	< .001
**Other laboratory findings**		
Aspartate aminotransferase (AST), U/L*	1.05 (1.03–1.07)	< .001
Alkaline phosphatase (ALP), U/L*	1.02 (1.01–1.03)	< .001
Lactate dehydrogenase (LDH), U/L*	1.02 (1.01–1.02)	< .001
gamma-Glutamyl transpeptidase (γ-GT), U/L*	1.0081 (1.0014–1.0148)	0.017
Total bilirubin, μmol/L*	1.11 (1.06–1.17)	< .001
Albumin, g/L*	0.75 (0.69–0.81)	< .001
Urea, mmol/L*	1.53 (1.33–1.77)	< .001
Uric acid, μmol/L*	1.0032 (1.0008–1.0055)	0.007
K^+^, mmol/L*	2.50 (1.53–4.08)	< .001
Ca^2**+**^, mmol/L*	0.26 (0.12–0.51)	< .001
High-sensitivity C-reactive protein (hs-CRP), mg/L*	1.02 (1.02–1.03)	< .001
Erythrocyte sedimentation rate (ESR), mm/h*	1.03 (1.02–1.04)	< .001
Blood platelet (PLT) count, × 10^9^/L*	0.9907 (0.9865–0.9950)	< .001
Prothrombin time (PT), s*	2.15 (1.67–2.78)	< .001
Activated partial thromboplastin time (APTT), s*	1.09 (1.03–1.14)	0.001
D-D dimer, μg/mL FEU*	1.33 (1.17–1.51)	< .001
**Multivariate analysis**		
Number of events/patients (%)	105/237 (44.3%)	…
Age, years*	1.10 (1.06–1.13)	< .001
Neutrophil/lymphocyte ratio*	1.14 (1.08–1.20)	< .001
Admission body temperature, °C*	1.53 (1.00–2.35)	0.005
AST: 1 (increase) vs. 0 (reference)	2.47 (1.16–5.26)	0.019
Total protein: 1 (decrease) versus 0 (reference)	1.69 (0.78–3.64)	0.018

*Continous variable; *AOR* Adjusted odds ratio, *CI*  Confidence interval.

### Construction of a prognostic model for vital status and survival in SARS-CoV-2

**Figure 1:**
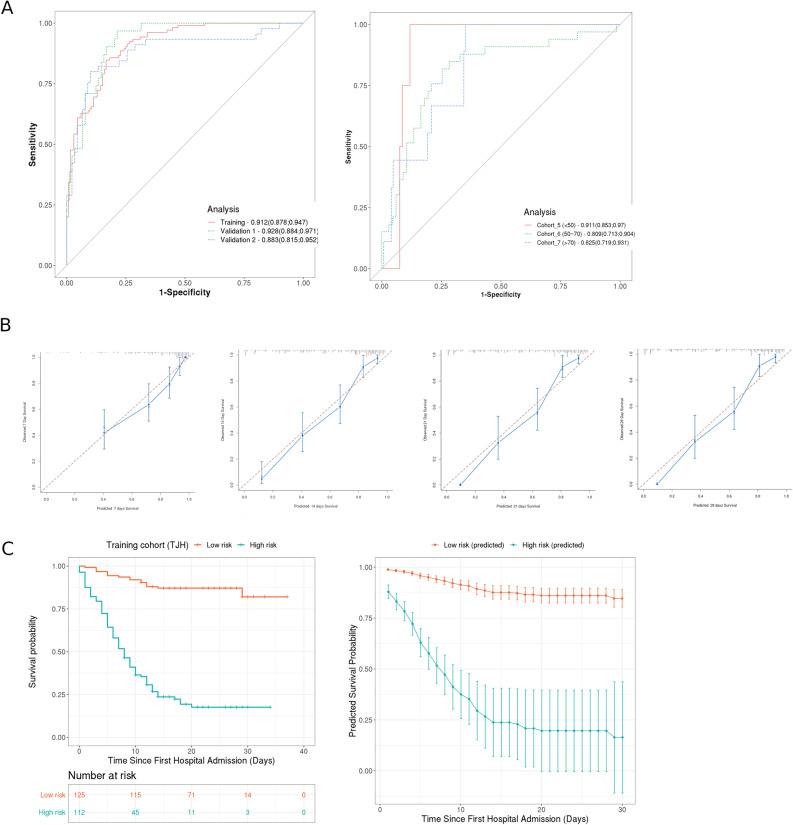
(**A**) AUC (area under curve) of the ROC (receiver operating characteristic) analysis for the training cohort and two validation cohorts (left), and for the age-specific three cohorts (< 50 year, 50–70 year, and > 70 year, right). (**B**) Calibration plot showed the comparison between predicted and observed survival rates of patients in training cohort on 7th, 14th, 21th, and 28th after hospital admission. (**C**) Clinical stratification and prediction of survival rate on the basis of the developed prognostic model. Survival in the low and high risk subgroups in training cohort (TJH) were stratificated by the a cutoff of ≤ 799 and > 799, respectively (left), predicted survival rates in the this cohort (right). Smooth lines represent mean predicted survival probabilities for each risk group; dots symbolize corresponding predicted rates with 95% CI (vertical lines) (this figure is produced using R version 3.6^37^).

For the TC, a multivariate analysis was performed to analyze the association between vital status, survival time, and all the covariates listed in Table 1. Five covariates were statistically significant predictors for vital status and survival time: (1) age (adjusted odds ratio (AOR): 1.1/years increase [95% CI 1.06–1.13]; Wald’s *p* < 0.001), (2) NLR (AOR: 1.14 increase [95% CI 1.08–1.2]; *p* < 0.001), 3) body temperature at admission (AOR: 1.53/°C increase [95% CI 1.0–5.26]; *p* = 0.005), 4) aspartate transaminase (AST) (AOR: 2.47 [95% CI 1.16–5.26] for increase vs. normal; *p* = 0.019), and 5) total protein (AOR: 1.69 [95% CI 0.78–3.64] for decrease vs. normal ; *p* = 0.018; Table 2). Based on the weights (coefficients) of these five significant covariates (Table 2), a prognostic model was constructed and applied to predict the vital status of the training cohort. The results of this analysis yielded an AUC of 0.912 (95% CI 0.878–0.947; Fig. 1A). This indicated that the prognostic model was able to effectively differentiate between patients with SARS-CoV-2 pneumonia who survive and were subsequently discharged versus those who died. In the prediction of overall survival, the model reached a Harrell’s c-index of 0.758 (95% CI 0.723–0.793; Fig. 1C). The model was also able to define a high-risk subgroup with a significantly increased likelihood of death due to SARS-CoV-2 pneumonia (hazard ratio [HR]: 24.22 [95% CI 10.57–55.5]) versus a low-risk subgroup. The predicted survival probabilities were compared with observed survival probabilities on the 7th, 14th, 21th, and 28th day after admission (Fig. 1B). The nomogram was constructed to assess impact of these factors (Supplementary Fig. 2). The predicted 30-days survival rates of the high- and low-risk subgroups in the training cohort are visualized in Fig. 1C (≥ 799 and < 799). Here, 799 represented the cutoff in the model based on the average of minimum calculated scores among deceased patients.

### Validation of the model for vital status and survival

**Figure 2:**
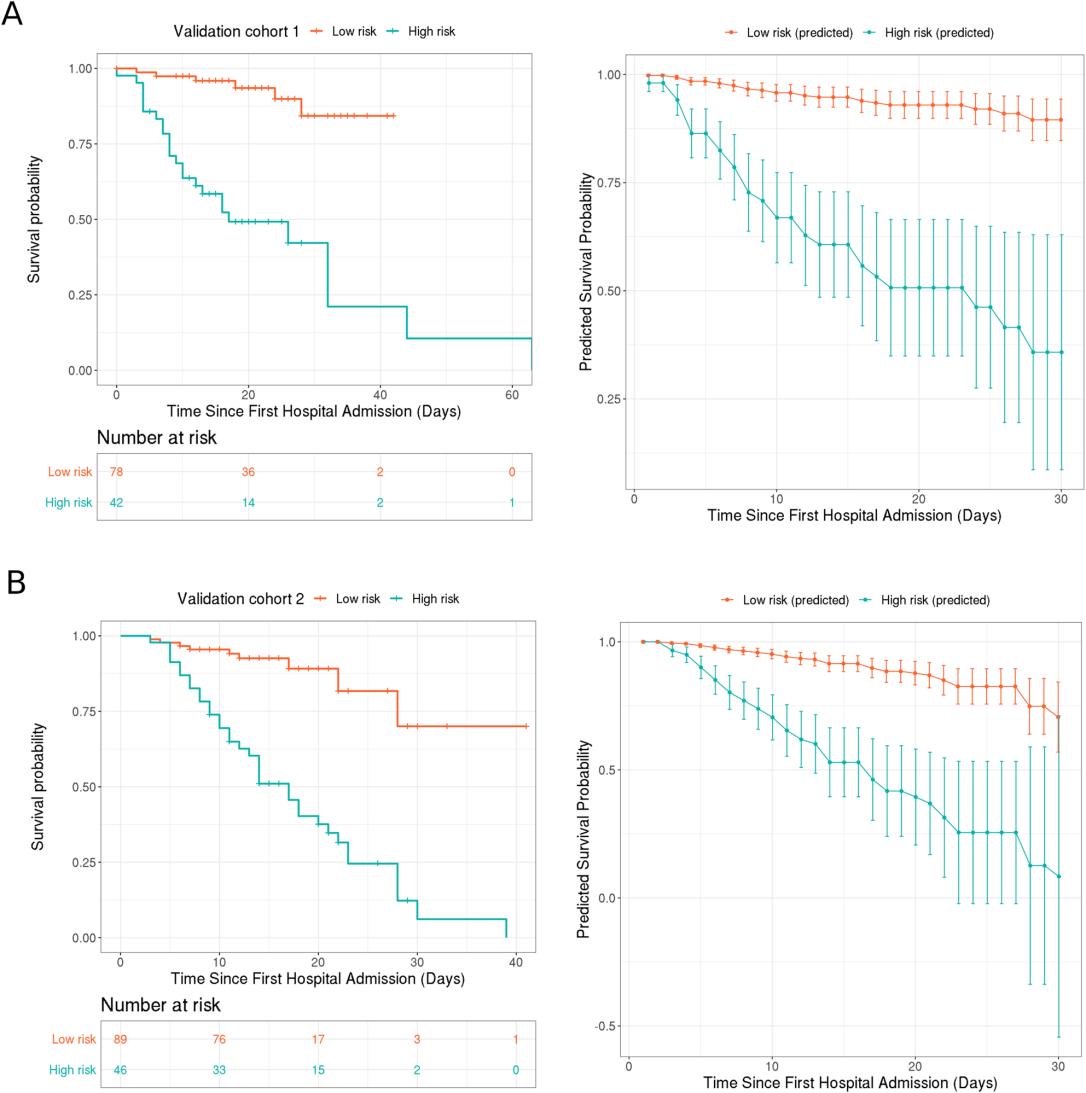
Prognostic model achieves clinical stratification and predicts overall survival (OS) in two validation cohorts. (**A**) Survival probabilities in the low- and high-risk subgroups defined by the consistent cutoff of 799 in the validation cohort 1 (RHWU + WNH) (left), correspondingly predicted survival probabilities in this cohort (right). (**B**) Survival probabilities in the low- and high-risk subgroups defined by the same cutoff in the validation cohort 2 (WPH) (left), correspondingly predicted survival probability in this cohort (right) (this figure is produced using R 3.6^37^).

In order to validate the prognostic value of the established outcome prediction model for SARS-CoV-2 pneumonia, external validation using 2 cohorts (VC1 and VC2) was performed. The model reached an AUC of 0.928 [95% CI 0.884–0.971; VC1] and 0.883 [95% CI 0.815–0.952; VC2] to predict the vital status (Fig. 1A). For the prediction of survival of both validation cohorts, the model yielded C indices of 0.762 [95% CI 0.723–0.801; validation cohort 1] and 0.711 [95% CI 0.672–0.75; validation cohort 2] (Fig. 2). By applying the same cutoff of model score, high-risk subgroups with lower survival rates were defined to clearly differentiate between the low-risk subgroups in both validation cohorts (HR: 11.53 [95% CI 4.01–33.15 for VC1 and HR: 9.3 [95% CI 3.32–26.03] for VC2) (Fig. 2). Of note, the predicted 30-day survival rates in high- and low-risk subgroups in both validation cohorts were similar to the observed survival rates in the training cohort (Fig. 2), thereby confirming the strength of the model for the prognosis for SARS-CoV-2 pneumonia.

To investigate the impact of age on the prognostic model, these two validation cohorts were merged and then divided into three groups by age to form three subgroups: < 50 year (cohort_5), 50–70 year (cohort_6), and > 70 year (cohort_7), respectively. For the prediction of the vital status, the model yielded an AUC of 0.911 [95% CI 0.853–0.97; cohort_5], 0.809 [95% CI 0.713–0.904; cohort_6], and 0.825 [95% CI 0.719–0.931; cohort_7; Fig. 1A]. For the survival prediction, the model yielded C indices of 0.572 [95% CI 0.533–0.611; cohort_5], 0.721 [95% CI 0.682–0.76; cohort_6], and 0.706 [95% CI 0.667–0.745; cohort_7; Table 3]. Finally, to aid in the current clinical management of SARS-CoV-2, a web-based application (http://82.165.167.23:8734/SIMTaskMaster/SARS2_Tool) was developed to enable broad testing and utilization of the developed prognostic model (Supplement Fig. 3).

**Table 3:** Vital status and overall survival prediction in age-specific cohorts.

Covariate	Coefficient	Score					
Age, years*	0.17	2 × Age (years)					
Neutrophil/lymphocyte ratio*	0.45	4 × Ratio					
Admission body temperature, °C*	1.73	17 × Temperature (°C)					
Aspartate transaminase (AST)	2.62	26 × (0/1; 0: reference, 1: increase)					
Total protein	2.71	30 × (0/1; 0: reference, 1: decrease)					
**Total computed score and risk stratification**							
Low risk	≤ 799						
High risk	> 799						

Cohort	Age (IQR)	No	Mortality (%)	AUC (95%CI)	C-index	*p *value	PM score
Cohort 5	39.0 (35.0–45.0)	97	4.1	0.91 (0.85–0.97)	0.572	0.050	785 (756–818)
Cohort 6	63.5 (57.8–66.0)	100	33.0	0.81 (0.71–0.90)	0.721	< .001	809 (777–841)
Cohort 7	77.5 (72.0–82.0)	58	67.2	0.83 (0.72–0.93)	0.706	< .001	856 (824–879)

*Continous variable; No. = Number of patients; AUC = Area under the curve; CI = Confidence interval; PM = Prognostic model.

## Discussion

In this retrospective multicenter study of 492 hospitalised patients with SARS-CoV-2 pneumonia, we found that advanced age, high body temperature on admission, high NLR, elevated AST as well as decreased total protein was associated with an increased risk of mortality. The prognostic model established based on these five clinical parameters was robustly validated using two separate validation cohorts. The aim of model application was the early identification and prioritization of individual patients requiring early administration of intensive treatment strategies.

The rapid transmission of the disease and the current second wave of COVID-19 pandemic have created public crisis on a global scale. To avoid overwhelming the public health systems and exacerbating the economic burdens, strategies to overcome this pandemic are being vigorously explored, including studies aimed at identifying the greatest at-risk populations. Prior studies have reported various potential risk factors associated with mortality in the setting of SARS-CoV-2 pneumonia^4,5^. For instance, Chen and colleagues found that age, obesity, and comorbidity were three identifiable risk factors for mortality^5^. Wang and colleagues identified that neutrophilia, lymphopenia, and elevated D-dimer and creatinine level were observed in non-survivors, implying that a cellular immune deficiency plus coagulation activation could potentially mediate disease severity^11^. In our study, the association between increased NLR and mortality suggests that altered immune cell function plays a critical role in the pathogenesis of SARS-CoV-2 pneumonia. This result is consistent with several recent independent studies^12–14^.

Advanced age has also been identified as an independent risk factor of COVID-19^5,15,16^. The underlying mechanisms could include changes of anatomical respiratory structure with aging^17^, immunosenescene^18^, and inflammaging^19^, which would, respectively, facilitate entry of SARS-CoV-2, weaken anti-viral immunity, and promote a cytokine storm, leading to multiple organ damages. Further, age-related alterations in metabolism are known to underlay changes in innate and adaptive immunity^20^, which also contribute to the weakening of immunity. Our findings that increased NLR in elderly patients is a major risk factor for mortality support the role of inflammaging in COVID-19 pathogenesis.

Aspartate transaminase (AST) is an important clinical marker for early diagnosis of various diseases including progression and/or metastatic potential of solid tumor^21,22^. Further, AST is an important enzyme involved in diverse metabolic pathways including purine metabolism^23^, steroid biosynthesis^24^, and synthesis of amino-acids such as arginine^25^, phenylalanine^26^, tyrosine^27^, and others^28^. Thus, elevated serum AST levels is considered an indicator of metabolic dysfunction. Furthermore, hypoalbuminaemia—manifested in our study as reduced total protein— is often related to malnutrition and recent studies have shown that diminished availability of metabolic nutrients directly leads to changes of immune responses^29–32^.

Because SARS-CoV-2 replication and pathogenesis are highly dependent on the host metabolism^33^. The decreased total protein strongly suggests a heightened viral burden and predicts a severe disease course. In total, poorer outcomes observed in our patients with elevated AST levels and decreased total protein could be related to age- and/or virus-induced metabolic dysfunction in these individuals.

Lastly, an increase in body temperature is one clinical manifestation of pro-inflammatory cytokine production (e.g., TNFα, TNFβ, IL-1β) by activated macrophages and T-lymphocytes. Dysregulated production of such cytokines can lead to a “cytokine storm” that ultimately damages vital organs, including the lungs, contributing to ARDS. Dysregulated and sustained TNFα production in response to other viral infections (HIV/AIDS) also mediates cachexia (muscle wasting) and is characterized by changes in total protein^32^. Thus, an elevated body temperature in patients at risk for mortality from COVID-19 pneumonia may reflect an aberrant cytokine response to SARS-CoV-2 infection.

In sum, these five clinical parameters, when combined, predict mortality in patients with COVID-19 pneumonia in our model are reflective of the status of host immunity. Further, as with other coronaviruses, SARS-CoV-2 does not possess its own metabolism, the viral replication and pathogenesis are highly dependent on the host metabolism. The hijack of host metabolism remains the only way for the viral survival.

Our study has several strengths. First, while several studies have previously reported relevant risk factors associated with SARS-CoV-2 pneumonia^34–36^, our study combined such factors into a robust and validated prognostic model for outcome of COVID-19 infection. Second, our model utilizes five commonly used clinical parameters that are routinely obtained on hospital admission and are not confounded by prior treatment since this has not yet been initialized. Third, our study involved a large number of patients and the prognostic model was fully validated with two large, independent external cohorts. Fourth, the model was also validated for age-specific cohorts. Specifically, the high AUC and C-indices of the prediction of the vital status and survival in patients aged 50–70 years versus > 70 indicate the suitability of the prognostic model for elderly patients. In total, this established prognostic model can assist clinicians in identification and stratification of high risk patients, thereby promoting initialization of vital treatment strategies that can improve outcomes.

The major limitation of this study is that the model was developed and validated purely based on Chinese population. Therefore, its application to the regions outside of China needs to be further determined. We speculate that the model could still reach a high prediction rate, however, the cutoff of optimal model score of 799 might need to be adjusted correspondingly to cover a broader spectrum of disease trajectories. Further, our prognostic model excluded gender and presence of comorbidity due to low statistical significance. One of reasons might be that this study was not able to include COVID-19 patients outside of China. Moreover, another study of our group has shown that presence of comorbidity was an age-dependent risk factor for COVID-19 infection (under review).

In addition, due to the nature of an observational study, potential confounders may exist which can have impacts on the results. Therefore, further prospective international multicenter studies are needed to test the robustness of this model.

## Conclusion

In this retrospective multi-center cohort study, a prognostic model was developed and validated to predict the outcome of individual patients suffering from SARS-CoV-2 pneumonia. We identified five common clinical parameters that are relevant to outcome of COVID-19 infection. This model enables clinical patient stratification to efficiently prioritize medical resources in the treatment and management of patients with SARS-CoV-2 pneumonia. The model’s clinical application may also inform treatment recommendations to save more lives in a high-risk group of patients while avoiding overtreatment in those at lower risk.

## Supplementary Information

Supplementary Legends.

Supplementary Figure 1.

Supplementary Figure 2.

Supplementary Figure 3.

## Data Availability

The data that support the findings of this study are available on request from the corresponding author.
