# Supplementary material for: Development and validation of prognostic model for predicting mortality of COVID-19 patients in Wuhan, China
Source: Sci Rep. 2020 Dec 31;10:22451. doi: 10.1038/s41598-020-78870-6 (PMC7775455; doi:10.1038/s41598-020-78870-6)
Supplement: Supplementary file 1 — Supplementary Legends. [file 41598_2020_78870_MOESM1_ESM.docx]

**Supplement Figure 1**. Study flow diagram. The three cohorts of this study.

**Supplement Figure 2**: Nomogram including age, body temperature by hospital admission, change level of aspartate transaminase, total protein, ratio of neutrophile-to-lymphocyte, and 7-, 14-, 21- and 28-day overall survival (OS) probabilities. This nomogram allows the user to obtain the probability of 7-, 14-, 21- and 14-days OS probabilities to a patient’s combination of covariates. Each covariate is associated with an influence degree (points) (this figure is produced using R 3.6 ^37^).

**Supplement Figure 3**: Individualized web-based Prediction Application.
