# Supplementary figures and images for: Development and validation of prognostic model for predicting mortality of COVID-19 patients in Wuhan, China
Source: Sci Rep. 2020 Dec 31;10:22451. doi: 10.1038/s41598-020-78870-6 (PMC7775455; doi:10.1038/s41598-020-78870-6)

# Supplement Figure 2. Nomogram Analysis

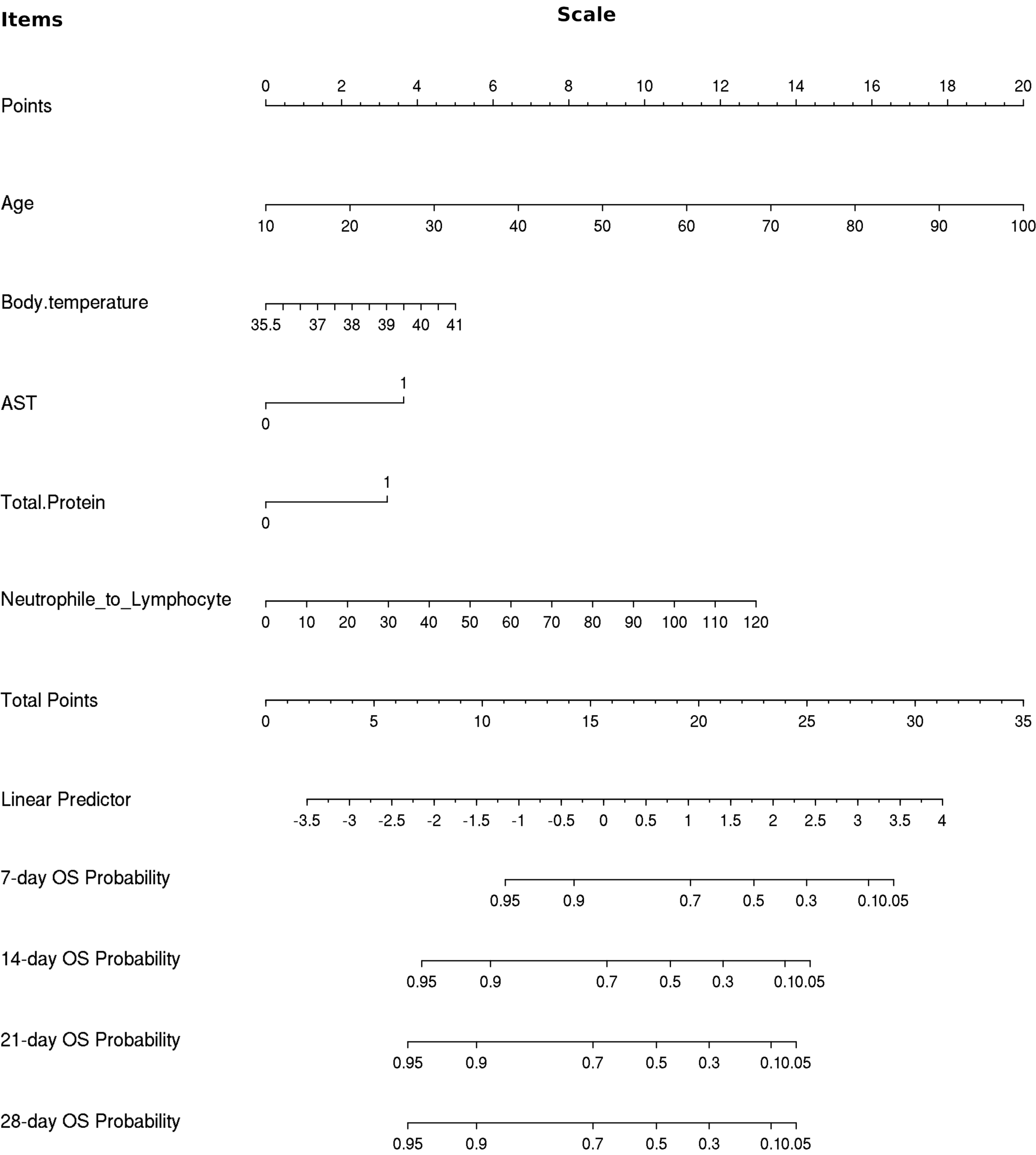

Supplement: Supplementary file 3 — Supplementary Figure 2. [file 41598_2020_78870_MOESM3_ESM.pdf]
