# Supplementary material for: Development and validation of prognostic model for predicting mortality of COVID-19 patients in Wuhan, China
Source: Sci Rep. 2020 Dec 31;10:22451. doi: 10.1038/s41598-020-78870-6 (PMC7775455; doi:10.1038/s41598-020-78870-6)
Supplement: Supplementary file 4 — Supplementary Figure 3. [file 41598_2020_78870_MOESM4_ESM.pdf]

Predict Survival-Status of SARS-CoV-2 Pneumonia

Age

Body temperature

Neutrophil-to-Lymphocyte Ratio

Total Protein Please specify

Aspartate transaminase Please specify

Predict
