# Supplementary material for: Development and validation of prognostic model for predicting mortality of COVID-19 patients in Wuhan, China
Source: Sci Rep. 2020 Dec 31;10:22451. doi: 10.1038/s41598-020-78870-6 (PMC7775455; doi:10.1038/s41598-020-78870-6)

Supplement Figure 1

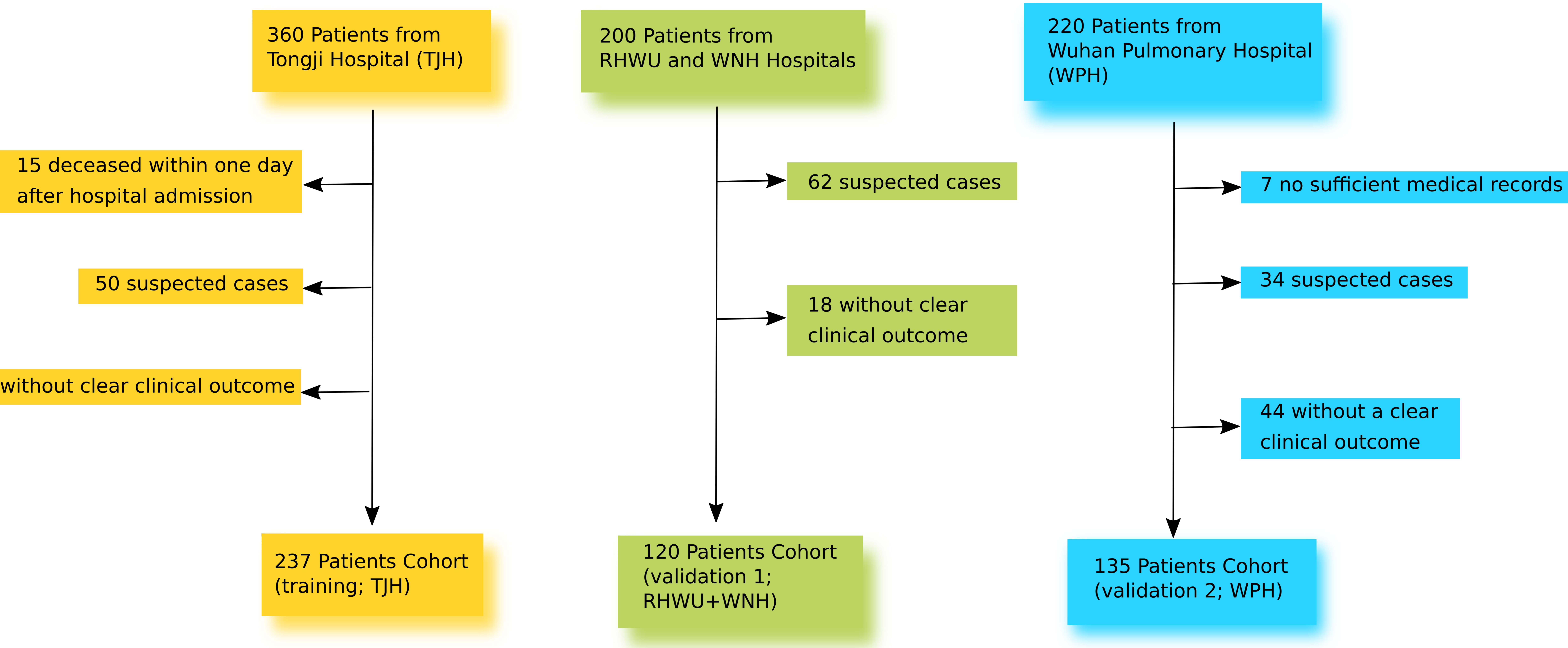

Supplement: Supplementary file 2 — Supplementary Figure 1. [file 41598_2020_78870_MOESM2_ESM.pdf]
